# Putative Mechanism of Action of Trazodone-Related Oromandibular Dyskinesia

**DOI:** 10.1155/2024/5543023

**Published:** 2024-03-31

**Authors:** Alan L. Schneider

**Affiliations:** University of Southern California Keck School of Medicine, Los Angeles, CA, USA

## Abstract

This is a case report of three cases of trazodone-induced buccal–lingual dyskinesias. Each case demonstrated the distinct pattern of the development of this dyskinesia after trazodone exposure for several months. All cases showed abrupt cessation of the movement disorder when the drug was discontinued. One of the three cases demonstrated a highly unusual presentation of an on/off pattern of buccal dyskinesia directly related to repetitive exposure and termination of the drug trazodone. Two of the three cases had no prior exposure to any dopamine blocking agents. One of the three had a distant exposure to a dopamine antagonist. As opposed to other antidepressants, trazodone has a mechanism of action which can account for both the development and treatment of dyskinetic movements. Its metabolite, M/chlorophenylpiperazine (M-CPP) is a 5HT2C agonist capable of causing abnormal oral-facial movements in rodent models. The presence of oromandibular dyskinetic movements can occur spontaneously with age, with trazodone being a potential predisposing factor. This article will discuss proposed mechanisms for trazodone's action with an emphasis on case reports of dystonic movements.

## 1. Introduction

Antidepressants are some of the more widely prescribed classes of drugs in Western countries. They have a broad base of therapeutic indications that range from depression to pain, anxiety, obsessive–compulsive disorder, and eating disorders. They act through the monoamine neurotransmitters serotonin and noradrenaline, and to a lesser extent dopamine. Reports of extrapyramidal symptoms with many antidepressants have been well-documented; however, relative to its high usage patterns as a sedative, little has been reported about the medication trazodone in terms of the development of buccal dyskinesias. The following three cases demonstrate trazodone's capacity to produce a reversal oral mandibular dyskinesia after relatively brief exposure. In each case, there was no evidence that other concurrent drug exposures could easily account for the development of this movement disorder.

## 2. Case Reports

1. This 69-year-old male was diagnosed with generalized anxiety disorder and longstanding sleep onset difficulties which were initially thought to be a function of anxiety. The patient began to complain of severe difficulty in both sleep onset and sleep maintenance. Intercurrent trials of eszopiclone 3 mg and suvorexant 10 mg then 20 mg, as well as patient-obtained lorazepam 2 mg were found to be ineffective for sleep maintenance. Trazodone was initiated at 50 mg and then raised to 100 mg, which was helpful for sleep maintenance. After 8 months, the dose was lowered to 50 mg. Concurrent treatment consisted of escitalopram 15 mg for generalized anxiety, evolocumab 140 mg SC Q2 weeks for hyperlipidemia, and amlodipine/valsartan 10/160 mg QD for hypertension. The patient's Epworth score was 11 prior to trazodone initiation and polysomnography showed severe obstructive sleep apnea with an apnea/hypopnea index of 32. Concurrent medical problems included hypertension, heart failure, and generalized anxiety.

After 4 months of trazodone exposure, the patient manifested a significant buccal dyskinesia noted as lip puckering at 3 s intervals. The patient himself had no awareness of this movement, although it disturbed his family. He had no prior exposure to antipsychotics, metoclopramide, lithium, or antidepressants other than those listed. Despite this, he was reluctant to discontinue the trazodone due to good response. On three separate occasions, he reinitiated trazodone treatment on his own with recurrence of the dyskinesia, followed by cessation when directed to stop the medication each time. Cessation took approximately 3–4 weeks on each occasion. In this case, the Naranjo Scale was 11, suggesting high concurrence and reproducibility of this adverse effect.

(2) This is a 65-year-old female with a history of recurrent major depression and alcohol use. The patient had a 10-year history of paroxetine 20 mg QD treatment for depression, followed by exposure to sertraline up to 150 mg for 8 years. Sertraline was augmented for a brief period (6 months) with low dose aripiprazole of 5 mg. No abnormal movements were detected during exposure to aripiprazole or for the year after discontinuation. Posttreatment for alcohol use disorder the patient was placed on trazodone 50 mg at night for sleep disruption, at which time the Epworth scale was 7. After 2 months of exposure, a lingual dyskinesia developed manifested by tongue thrusting toward the palate approximately 6 or more times per minute. After cessation of trazodone, the movements ceased within 3 weeks. The Naranjo Scale was 8.
(3) This 37-year-old married female originally presented with major depression and obsessive–compulsive disorder of mild intensity. The patient had an initial 8 years of exposure to citalopram 40 mg followed by 3 years of sertraline 100 mg. Her course was complicated by the diagnosis of chronic fatigue syndrome around the age of 36. Comprehensive laboratory panels showed an ANA elevated at 1 : 80 with a homogeneous pattern, normal c-reactive protein and sedimentation rate, and an elevated rheumatoid factor of 17.5. During the course of treatment, sleep fragmentation was treated with trazodone 50 mg for 2 months. The patient herself noticed the emergence of a buccal–lingual dyskinesia manifested by puckering and tongue protrusion multiple times per minute, which she found quite distressing. She had no intercurrent exposure to dopamine agonists or antagonists. Rapid withdrawal of the trazodone resulted in a cessation of symptoms almost immediately. The Naranjo Scale was 7.

## 3. Discussion

Hyperkinetic movement disorders are usually associated with chronic use of dopamine blocking agents. Oromandibular dyskinesias (OMD) can be acquired, inherited, or idiopathic in nature [1, 2]. They are noted to be related to increasing age as well as mass lesions in the CNS, arteriovenous malformations, toxins, or hemorrhagic injury. OMD can be either acute or chronic, often dependent upon the length of exposure to an offending agent as well as the patient's age.

Tardive dyskinesia refers to late onset abnormal involuntary movements that are purposeless in nature. These most often effect the tongue, mouth, face, and limbs. Most classically, the disorder is thought to be associated with antipsychotic exposure for at least three continuous months with prevalence rates as high as 30% [2, 3].

The mechanism for drug-induced movement disorders is classically thought to be drug induced occupancy of D2 receptors with related postsynaptic supersensitivity of these same receptors. This can then result in the disinhibition of the globus pallidum internus and subthalamic nucleus, which can result in hyperkinetic syndromes [3]. It has also been observed that extrapyramidal movements have been associated with serotonin reuptake inhibitor medications however at a very low prevalence rate [4, 5]. The mechanism for this is thought to be antagonism of 5HT2 receptors in GABAergic interneurons of nigrostriatal pathways. This action can suppress the activity of dopaminergic neurons leading to such movements [6].

Preclinical studies in rodents have demonstrated that 5HT2C receptor stimulation or blockade produces abnormal oral-facial responses. Classically 5HT2C agonists have been shown to inhibit dopamine neuronal activity, which may be one pathway for the release of abnormal motor movements [7].

Serotonin 5HT2C receptors are one of seven transmembrane G-coupled receptors which play a role in neuronal excitability. It works in three states, that of phasic, tonic, and constitutive activity initiated by agonists and ablated by inverse agonists. These receptors exert tight control over oral facial activity through alteration of aphasic and constitutive control of the 5HT2 receptor and can in fact promote purposeless oral movements through the basal ganglia. 5HT2C agonists inhibit dopamine triggered behaviors [6].

Further evidence for the association between animal and human effect of the 5HT2C receptor is found in the polymorphisms of the 5HT2C23 Ser allele, which predisposes male schizophrenic patients to have an increased level of developing extrapyramidal symptoms [8].

Trazodone is a triazolopyridine 5HT2A and 5HT2C receptor antagonist class of antidepressant. It also possesses antagonist effects at the alpha 1A and 2A adrenoreceptors, and H1 histaminergic receptors which accounts for its sedating profile. Its off-label uses have included treatment of insomnia, delirium, and dystonias [9]. It was introduced in the United States in 1978 and is considered to be low in anticholinergic properties with limited effect on cardiac induction [10]. It is greater than 90% protein bound, extensively metabolized by the 3A4 hepatic enzymatic system, and is 75% excreted through urine. Its metabolite m-chlorophenylpiperazine (MCPP) has a ½ life of 4–14 hr with no significant affinity for dopaminergic receptors. However, MCPP is known to be a serotonin agonist specifically at the 5HT2C receptor that may have a releasing effect on oral-facial abnormal movements [11]. There are competing models suggesting that trazodone use could result in the development of OMD. Studies conducted in rat models suggest that trazodone can in fact block postsynaptic striatal dopamine receptors. This aspect of the drug may account for its capacity to develop dyskinetic movements [12, 13].

An alternate concurrent theory is that trazodone may trigger extrapyramidal systems through inhibitory action on T-type calcium channels in the subthalamic nucleus, an effect seen in first-generation antipsychotics [14]. Additionally, the case that 5HT2A receptor polymorphisms determine susceptibility to dyskinesias/dystonias with antidepressants. It is unknown if this effect is more prominent in the older population [11, 13, 15]. In this case series two of the three patients were in their 60s which raises the question of whether older individuals who may be more prone to age-related dyskinesias may be more sensitive to the trazodone metabolite associated with hyperkinetic syndromes. Other case reports have seen trazodone used successfully to treat tardive dyskinesia, dystonia, and akathisia produced by antipsychotics [16, 17].

Two of the three cases involved individuals over the age of 65. In one of these two, symptoms persisted for weeks after discontinuation of the medication. Drug-induced movement disorders in the elderly are an important subgroup of movement disorders. Classically, these have been restricted to dopamine receptor blockers, serotonin reuptake inhibitors, opioids, and some anesthetic agents like propofol and morphine. There may be higher incidence of dyskinesia with age given one theory that metabolic inactivation of medications lowers with increasing age leading to increase in medication sensitivity. In one of the two older patients the effect of age plus history of excessive alcohol use could very well predispose to the development of a movement disorder postexposure to trazodone. In the other older patient, similarly age plus a series of medical comorbidities including obstructive sleep apnea, hypertension, and heart failure may play a role in increasing the sensitivity to trazodone-induced dyskinesia [18]. Risk factors in the younger patient cited are not as clear; however, the presence of obsessive–compulsive disorder does have relationship to motor disorders such as tics and could in fact predispose to the development of other movement disorders [19].

## 4. Conclusion

Many antidepressants have been associated with the development of hyperkinetic syndromes, although attention is most commonly focused on exposure to first- and second-generation antipsychotics with their known D2 blocking activity. In reality, these syndromes may be more multifactorial in nature.

Trazodone has been used in the psychiatric and primary care sectors for a number of off-label applications, most prominently for sleep maintenance. This case series suggests that more consideration should be given to the medication's capacity to induce OMD. Therefore, further surveillance is indicated.

## Data Availability

Data supporting this study are available on request.
